# Patterns of IgG and IgM antibody response in COVID-19 patients

**DOI:** 10.1080/22221751.2020.1773324

**Published:** 2020-06-09

**Authors:** Xuemei Liu, Jing Wang, Xiaolei Xu, Guojian Liao, Yaokai Chen, Chang-Hua Hu

**Affiliations:** a College of Pharmaceutical Sciences, Medical Research Institute, Southwest University, Chongqing, PR People’s Republic of China; b Division of Medical Laboratory Sciences, Chongqing Public Health Medical Center (Southwest University Public Health Hospital), Chongqing, PR People’s Republic of China; c Division of Infectious Diseases, Chongqing Public Health Medical Center (Southwest University Public Health Hospital), Chongqing, PR People’s Republic of China

## To the editor

Coronavirus disease 2019 (COVID-19), which emerged in Wuhan, China in December 2019, is caused by severe acute respiratory syndrome coronavirus 2 (SARS-CoV-2) and has become a major global public health concern [1]. Positive detection of SARS-CoV-2 RNA in nasopharyngeal swab samples, sputum samples or bronchoalveolar lavage samples by reverse transcriptase polymerase chain reaction (RT–PCR) has been used to confirm SARS-CoV-2 infection [2]. Recently, positive detection of IgM and IgG antibodies specific to SARS-CoV-2 has also been recognized as deterministic evidence for confirmed SARS-CoV-2 infection [3,4]. However, the antibody response to SARS-CoV-2 currently remains inadequately understood in COVID-19 patients. In the present study, we investigated the patterns of antibody response to SARS-CoV-2 in patients with COVID-19, aiming to better clarify the humoral immunological response during SARS-CoV-2 infection.

## Patient characteristics

A total of 32 patients with a confirmed diagnosis of COVID-19 were included in our cohort. All were positive for SARS-CoV-2 according to nucleic acid testing by RT–PCR of nasopharyngeal swab, sputum, or bronchoalveolar lavage specimens. Patients exhibiting one or more of the following conditions were classified as having severe COVID-19: (a) respiratory distress (≥30 breaths/min); (b) oxygen saturation ≤93% at rest; (c) arterial partial pressure of oxygen (PaO_2_)/fraction of inspiration O_2_ (FiO_2_) ≤300 mmHg (1 mmHg = 0.133 kPa); (d) respiratory failure requiring mechanical ventilation; (e) septic shock development; or (f) critical organ failure requiring ICU care. Patients not meeting the above criteria were classified as having mild COVID-19.

The median age of the 32 patients was 55 years old, and 66.7% of them were male. Among the 32 patients, 18 (56.3%) were severe cases, and 14 (43.7%) were mild cases. The most common symptoms at onset of illness were fever, cough, fatigue, dyspnoea and headache. The demographic details and clinical disease severity of all patients in our cohort are shown in the supplementary materials (Table 1). Our study was approved by the Ethics Committee of Chongqing Public Health Medical Center (No. 2020-006-01), and informed consent was obtained from all subjects prior to blood sample collection.

**Table 1.** Demographic characteristics and antibody titre of 32 COVID-19 patients.

Patient number	Age	Sex	severe case	Sample number	ID	day	IgG (RU/ml)	IgM (RU/ml)
1	73	male	YES	2001278733	74827	4	0.94	15.57
				2001312952	74827	8	177.44	176.69
				2002059960	74827	13	664.2	282.74
				2002093544	74827	17	731.94	314.94
				2002127130	74827	20	665.58	236.87
2	52	male	YES	2001267752	74833	7	39.92	129.6
				2001290545	74833	10	189.55	194.99
				2002048959	74833	16	202.85	460.93
				2002093481	74833	21	246.72	632.64
				2002127090	74833	24	233.22	464
3	70	male	NO	2001299883	74839	10	98.27	11.88
				2001313114	74839	12	874.23	44.57
				2002059503	74839	17	1572.32	67.32
				2002093351	74839	21	1377.84	66.91
				2002140361	74839	26	1213.54	58.79
4	70	female	YES	2001267391	74851	7	0.97	2.1
				2001288808	74851	9	1.17	6.75
				2001312862	74851	12	66.3	25.02
				2002036073	74851	15	238.5	43.74
				2002060585	74851	18	275.85	94.55
				2002093998	74851	21	406.59	114.09
5	65	female	YES	2001267790	74862	7	2.43	13.9
				2001278746	74862	8	43.98	33.59
				2001299874	74862	10	74.37	109.79
				2001301290	74862	11	167.51	81.5
				2001313232	74862	12	299.69	124.98
				2002036823	74862	15	599.44	181.03
				2002072533	74862	19	535.16	132.12
				2002094013	74862	21	612.32	106.51
				2002116878	74862	23	596.47	104.57
				2002127664	74862	24	517.07	88.47
				2002139300	74862	25	674.04	115.42
				2002140505	74862	26	655.19	99.52
6	38	male	YES	2001267654	74863	9	21.43	50.74
				2001290540	74863	12	375.96	216.74
				2002013601	74863	15	585.37	223.08
				2002048134	74863	18	784.3	195.61
				2002072220	74863	21	692.54	188.64
				2002127169	74863	26	672.38	143.25
7	60	female	NO	2001278322	74877	3	0.93	7.14
				2001301110	74877	6	0.99	11.73
				2002013575	74877	8	2.74	27.07
				2002059329	74877	12	548.55	164.95
				2002093476	74877	16	1067.37	255.72
8	38	male	YES	2001289774	74912	9	83.75	114.35
				2001290543	74912	10	141.4	145.23
				2001301919	74912	11	190.77	184.79
				2002025775	74912	14	213.92	259.38
				2002048717	74912	16	187.13	344.38
				2002071492	74912	19	199.88	408.83
				2002127144	74912	24	221.51	491.04
9	56	female	YES	2001290419	74921	4	0.93	5.06
				2001313538	74921	6	2.78	8.81
				2002014606	74921	7	2.63	18.31
				2002061270	74921	12	21.88	329.72
				2002082793	74921	14	68.72	373.78
				2002104121	74921	16	127.14	436
10	55	female	NO	2001290328	74929	9	194.35	232.74
				2002013579	74929	12	266.34	233.96
				2002047843	74929	15	324.93	259.5
				2002071490	74929	18	350.07	283.65
				2002105698	74929	21	330.38	250.95
11	67	male	NO	2001290929	74935	6	1.03	17.41
				2002014497	74935	9	0.93	49.75
				2002025405	74935	10	4.37	115.26
				2002047701	74935	12	6.34	113.27
				2002072412	74935	15	199.16	193.81
12	38	male	YES	2001290938	74940	3	0.93	0.93
				2002014667	74940	6	0.93	3.91
				2002048327	74940	9	1.52	32.12
				2002083247	74940	13	380.16	144.28
				2002105333	74940	15	345.5	167.62
				2002116806	74940	16	438.22	134.65
				2002138679	74940	18	458.95	159.62
13	50	male	YES	2001301499	74948	10	197.05	186.68
				2002013584	74948	12	342.23	282.93
				2002047837	74948	15	437.01	354.36
				2002071486	74948	18	392.84	440.22
				2002105692	74948	21	352.06	380.02
				2002140348	74948	25	408.94	379.38
14	69	female	YES	2001301775	74952	2	0.93	0.93
				2001313338	74952	3	0.93	1.9
				2002037655	74952	6	2.37	17.23
				2002050036	74952	8	38.18	53.33
				2002072543	74952	10	105.89	49.7
				2002083134	74952	11	95.69	65.68
				2002093971	74952	12	87.47	61.8
				2002116847	74952	14	203.35	63.8
				2002127670	74952	15	217.68	68.85
				2002140610	74952	17	292.36	73.84
15	46	female	NO	2001301957	74959	3	1.25	2.66
				2002025001	74959	6	0.93	4.72
				2002037387	74959	7	1.59	9.16
				2002071617	74959	11	2.49	36.24
				2002116038	74959	15	40.35	64.59
				2002173413	74959	21	150.88	44.96
				2002239965	74959	27	194.04	32.56
				2002247400	74959	28	193.16	38.1
16	47	male	NO	2001302006	74965	10	17.94	51.54
				2002025482	74965	13	171.21	127.42
				2002059579	74965	16	333.47	238.26
				2002083197	74965	19	386.43	266.42
				2002140397	74965	25	401.4	275.64
17	29	male	NO	2001302051	74966	2	0.93	0.93
				2002037412	74966	6	0.93	3.9
				2002083093	74966	11	2.17	26.96
				2002128004	74966	15	2.35	59.39
18	59	male	YES	2001302104	74972	6	0.93	6.72
				2002025853	74972	9	0.93	3.06
				2002048964	74972	11	0.93	5.94
				2002071517	74972	14	118.25	24.53
				2002105336	74972	17	640.9	32.06
				2002138710	74972	20	940.68	32.43
				2002161517	74972	23	938.8	9.9264
				2002172344	74972	24	840.87	15.136
				2002206637	74972	27	936.13	15.8576
				2002212014	74972	28	704.64	15.5848
19	29	male	NO	2001312176	74976	7	0.96	1.36
				2002024985	74976	9	0.93	10.36
				2002059778	74976	12	1.54	60.06
				2002083023	74976	15	5.75	173.2
				2002127421	74976	19	14.51	264.95
20	64	male	NO	2001312184	74977	3	0.93	1.98
				2002024934	74977	5	0.93	29.85
				2002048795	74977	7	44.59	143.65
				2002060346	74977	9	357	283.46
				2002093320	74977	12	920.51	356.17
				2002127291	74977	15	914.47	307.65
				2002173315	74977	20	1172.36	107.26
				2002206672	74977	23	999.36	90.26
				2002247458	74977	27	641.88	86.3
21	55	male	NO	2002014486	75021	7	0.96	7.76
				2002059168	75021	11	1.46	10.33
				2002083123	75021	14	1.99	14.39
				2002138386	75021	19	136.11	107.79
				2002148386	75021	20	151.39	88.49
				2002183964	75021	24	168.56	71.72
22	57	female	NO	2002048799	75024	5	0.94	0.93
				2002071864	75024	8	1.46	9.03
				2002115816	75024	12	4.87	23.02
				2002128125	75024	13	0.93	8.05
				2002140291	75024	15	7.5	23.9
				2002150771	75024	16	6.32	9.6448
				2002162072	75024	17	20.44	11.1848
				2002172124	75024	18	46.43	12.5752
				2002185056	75024	19	64.48	13.6136
				2002196373	75024	20	95.51	11.6952
				2002206593	75024	21	112.44	15.8928
				2002218177	75024	22	98.31	13.0592
				2002229264	75024	23	110.88	12.496
				2002252541	75024	26	101.86	12.0296
				2002276138	75024	28	92.73	9.7768
23	43	female	YES	2002014480	75025	3	0.93	4.83
				2002061352	75025	8	1.03	4.36
				2002093995	75025	11	0.93	4.86
				2002127609	75025	14	21.46	46.51
				2002149871	75025	16	216.1	165.02
				2002183780	75025	20	623.16	98.78
24	36	male	YES	2002025485	75054	4	0.93	9.72
				2002037670	75054	5	0.93	26.65
				2002059680	75054	7	32.55	125.39
				2002072539	75054	9	179.46	280.55
				2002116856	75054	13	316.22	292.07
				2002140612	75054	16	569.92	184.53
25	42	male	YES	2002025637	75062	6	0.93	4.86
				2002059116	75062	9	0.93	4.6
				2002072520	75062	11	0.93	13.72
				2002093817	75062	13	10.87	56.42
				2002127491	75062	16	173.85	186.24
26	64	male	NO	2002025704	75063	3	0.93	2.42
				2002036781	75063	4	0.93	0.93
				2002059307	75063	6	0.93	7.35
				2002093337	75063	10	0.93	15.56
				2002149720	75063	15	1	9.37
27	51	male	YES	2002025573	75064	5	1.18	5.86
				2002050025	75064	8	1	6.89
				2002083137	75064	11	0.94	40.17
				2002127652	75064	15	151.37	214.94
				2002151216	75064	18	235.79	117.63
				2002207684	75064	23	200.61	98.39
				2002256162	75064	28	268.33	64.29
28	51	male	NO	2001313503	75067	7	3.95	23.07
				2002025616	75067	9	1.57	29.21
				2002059132	75067	12	64.59	85.86
				2002104233	75067	17	489.38	112.11
				2002149377	75067	21	158.84	155.96
29	63	female	NO	2002025748	75070	5	0.93	0.93
				2002059563	75070	8	0.93	4.74
				2002071702	75070	10	1.69	39.62
				2002093416	75070	12	25.16	100.78
				2002140375	75070	17	653.13	282.11
30	77	male	YES	2002036834	75091	7	1.74	6.88
				2002059179	75091	9	0.93	13.38
				2002061322	75091	10	1.07	14.8
				2002082791	75091	12	2.52	32.63
				2002104575	75091	14	4.07	47.88
				2002138576	75091	17	23.38	95.77
31	57	male	YES	2002048509	75131	4	0.93	2.46
				2002072082	75131	7	2.45	15.9
				2002093308	75131	9	1.41	8.07
				2002105687	75131	10	2.48	21.97
				2002116802	75131	11	3.23	29.59
				2002127853	75131	12	9.63	26.04
				2002138971	75131	13	15.94	35.01
				2002150964	75131	15	22.64	21.24
				2002184149	75131	18	50.6	30.84
				2002229495	75131	22	167.98	57.87
				2002240640	75131	24	213.22	53.94
32	68	male	YES	2002048842	75138	4	2.41	4.26
				2002061329	75138	6	0.93	10.52
				2002083162	75138	8	3.28	23.33
				2002093836	75138	9	4.19	34.83
				2002127457	75138	12	39.23	69.07
				2002161407	75138	16	126.36	43.64
				2002195400	75138	19	209.24	39.59
				2002230157	75138	23	157.06	28.29
				2002288414	75138	28	229.09	8.66

## Detection of antibodies against SARS-CoV-2

In total, 217 blood specimens were obtained from 32 patients (6.8 blood specimens per patient on average; supplementary materials). A quantum dot immunofluorescence assay was used to semi-quantitatively detect IgM and IgG antibodies. The anti-SARS-CoV-2 IgG and IgM kits were manufactured by Chongqing Xinsaiya Biotechnology Company in Chongqing, China. Assays were performed according to the manufacturer’s detailed instructions. Briefly, serum collected from patients was incubated at 56°C for 30 min, and then, 80 μl of the diluted serum was added to the well dented on the test chip and was incubated at room temperature for 10 min. During the process, IgM or IgG antibodies in the serum sample reacted with quantum dot nanocrystal-conjugated secondary antibodies and purified recombinant SARS-CoV-2 spike (S) protein, respectively, which were both coated on a cellulose nitrate membrane. Subsequently, the immunofluorescence signal strength of the sample was analysed by a quantum dot fluorescence detector, which emitted a wavelength of 610 nm and excited a wavelength of 365 nm. The quantitative results were expressed in relative vitality units (RU/ml) according to the calibration curve. A value ≥10 RU/mL was considered to be a positive result. All serum samples were tested in triplicate, and the average of all three relative vitality units was used as the final test result.

## Patterns of anti-SARS-CoV-2 IgG and IgM antibodies

**Figure 1.**
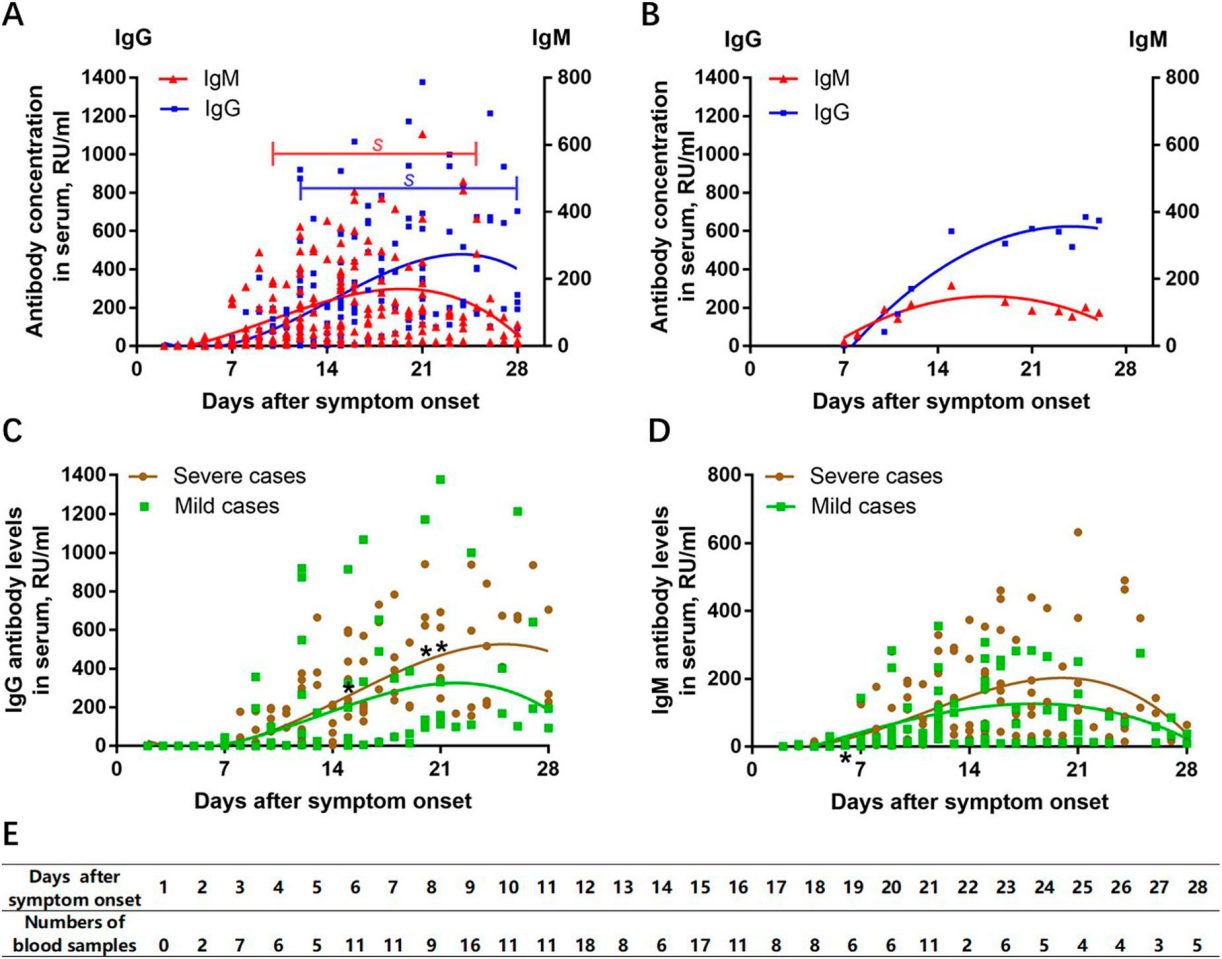
Patterns of anti-SARS-CoV-2 S antibody response in patients with COVID-19. (A) IgG and IgM antibody response patterns in serum samples of all 32 patients with confirmed COVID-19; *s*, a significant difference by non-parametric repeated measures ANOVA; (B) IgG and IgM antibody response in a 65-year-old woman with severe COVID-19; (C) The difference in anti-SARS-CoV-2 IgG antibody response between severe cases and mild cases; (D) The difference in anti-SARS-CoV-2 IgM antibody response between severe cases and mild cases. (E) Numbers of blood samples collected during the study period. For statistical analyses, the Mann-Whitney U test was performed for continuous variables. Statistical testing could not be performed on days 22, 24, 25 and 27 due to the very small number of available samples. *p < 0.05 was considered statistically significant.

As shown in Figure 1, anti-SARS-CoV-2 S-specific IgG and IgM antibodies were not detectable in the very early days of infection (from day 0 to day 3). Anti-SARS-CoV-2 S-specific IgM antibodies were detectable from day 4 onward; the IgM antibody titres increased over time, peaking at approximately day 20, and then began to decline. The positivity rate of IgM antibody was only 60%, with a marked reduction in antibody levels 4 weeks after onset of illness. Anti-SARS-CoV-2 S-specific IgG antibodies were identifiable from day 7 onwards, peaking at approximately day 25, as shown in Figure 1(A). Serum IgG antibodies were still maintained at a high level after 4 weeks of infection. Figure 1(B) shows a typical IgG and IgM antibody response in a 65-year-old woman with COVID-19 (supplementary materials, Table 1). It is widely accepted that the IgM antibody response provides early-stage defence during viral infections prior to the development of the class-switched, high-affinity IgG response for long-term immunity and immunological memory [5].

## Comparison of antibody response between mild cases and severe cases

We further compared the difference in antibody detectability between mild cases and severe cases of COVID-19. As shown in Figure 1(C), serum IgG antibody levels were not significantly correlated with clinical severity in the early stage of infection. However, the difference in IgG antibody levels between mild cases and severe cases from day 15 onward was found to be statistically significant (day 15 (*N* = 17), day 20 (*N* = 6) and day 21 (*N* = 11), all *p* < 0.05). Severe cases of COVID-19 tended to have a more vigorous IgG response against SARS-CoV-2 compared with mild cases. Notably, some patients with mild disease had a robust IgG antibody response from 9 days after symptom onset, while a few mild cases did not generate adequate IgG antibodies (approximately 21.43%). Our results also showed that mild cases tended to develop faster peak anti-SARS-CoV-2 S-specific IgM responses (approximately 17 days after symptom onset) compared with severe cases (approximately 21 days after symptom onset). It is also worth noting that IgM antibodies disappeared 4 weeks after symptom onset both in mild cases and severe cases (Figure 1(D)).

In summary, we observed that (1) the IgM antibody response to SARS-CoV-2 occurred earlier and peaked earlier than the IgG antibody response; (2) the IgM antibody response began to decline at week 3 of the illness, while the IgG antibody response persisted and was maintained in patients with COVID-19; and (3) severe cases of COVID-19 tended to have a more vigorous response in both IgG and IgM antibodies to COVID-19 illness. Our findings may be of significance in interpreting anti-SARS-CoV-2 antibody test results and in understanding humoral immune response patterns for SARS-CoV-2 infection in current and potential future COVID-19 outbreak scenarios. Importantly, the timing of IgM and IgG antibody occurrence in patients varies greatly, and this variation in timing may be associated with age as well as comorbidity [6]. More care needs to be taken when using levels of anti-SARS-CoV-2 antibodies to make a clinical diagnosis of COVID-19 or determine discharge criteria.
